# Conformational Plasticity Enhances the Brain Penetration of a Metabolically Stable, Dual-Functional Opioid-Peptide CycloAnt

**DOI:** 10.3390/ijms252111389

**Published:** 2024-10-23

**Authors:** Yangmei Li, William E. Cotham, Abbe Eliasof, Kathryn Bland, Michael Walla, Perry J. Pellechia, Chongguang Chen, Daping Fan, Jay P. McLaughlin, Lee-Yuan Liu-Chen

**Affiliations:** 1 College of Pharmacy, University of South Carolina, Columbia, SC 29208, USA; 2 Mass Spectrometry Facility, University of South Carolina, Columbia, SC 29208, USA; 3 Center for Substance Abuse Research, Temple University, Philadelphia, PA 19140, USAlee-yuan.liu-chen@temple.edu (L.-Y.L.-C.); 4 Nuclear Magnetic Resonance Facility, University of South Carolina, Columbia, SC 29208, USA; 5 School of Medicine, University of South Carolina, Columbia, SC 29209, USA; daping.fan@uscmed.sc.edu; 6 College of Pharmacy, University of Florida, Gainesville, FL 32610, USA; jmclaughlin@cop.ufl.edu

**Keywords:** cyclic peptide, opioid ligand, conformational plasticity, membrane-permeable peptide

## Abstract

CycloAnt is an opioid peptide that produces potent and efficacious antinociception with significantly reduced side effects upon systemic administration in mice. To verify its CNS-mediated antinociception, we determined its binding affinity at the opioid receptors, its proteolytic stability in mouse serum, metabolic stability in mouse liver microsomes, and pharmacokinetics in mice. CycloAnt exhibited stability toward proteolytic degradation in serum and resistance against metabolism mediated by cytochrome P450 enzymes (CYP450s) and UDP-glucuronosyl transferases (UGTs) in mouse liver microsomes. A pharmacokinetic study of CycloAnt in mice confirmed that CycloAnt crossed the blood–brain barrier (BBB) with a brain-to-plasma ratio of 11.5%, a high extent of BBB transport for a peptide. To elucidate the structural basis underlying its BBB penetration, we investigated its conformation in water and DMSO using ^1^H NMR spectroscopy. The results show that CycloAnt displays an extended conformation in water with most amide NHs being exposed, while in less polar DMSO, it adopts a compact conformation with all amide NHs locked in intramolecular hydrogen bonds. The chameleonic property helps CycloAnt permeate the BBB.

## 1. Introduction

Current morphinan-based prescriptive opioid pain medications, while effective analgesics, are associated with harmful side effects, including respiratory depression, tolerance, dependence, and abuse. Use and misuse of prescriptive opioid pain medications initiated the opioid-overdose crisis, which later has been fueled by illicit manufactured synthetic opioid fentanyl to become the third leading cause of deaths in the USA [1]. While some painkillers target alternate mechanisms to produce analgesia, they, too, produce adverse effects such as sedation, locomotor impairment, and possible habitual use [2,3]. Their efficacy often falls short of traditional opioids as well [4]. The urgent need for developing a safe, nonaddictive, highly efficacious analgesic holds the key to addressing the devastation of opioid misuse while improving pain management outcomes.

The endogenous opioid system uses opioid peptides to interact with the mu (MOR), delta (DOR), and kappa (KOR) opioid receptors. Over 20 endogenous opioid peptides work together to activate all three opioid receptors to effectively relief pain without producing adverse effects [5]. This differs dramatically from the small-molecule opioid pain medications. In addition, peptides and small-molecule drugs use different signaling mechanisms to activate the opioid receptors in the intracellular level. Following the initial receptor activation at the plasma membrane, opioid peptides propagate the receptors to endosomal activations. In contrast, small-molecule drugs, in addition to activating receptors at plasma membrane and endosomes, readily penetrate the cell membrane to drive the internal activation into the Golgi apparatus [6]. The spatiotemporal specificity affects signal duration and pathway selection downstream, contributing to distinct downstream physiological effects. Peptide-based opioid analgesics have, therefore, gained profound interest in the discovery and development of safer, highly efficacious, and non-/less-addictive analgesics [7,8,9,10,11,12].

The challenge in developing peptide-based drugs is always their poor proteolytic stability and low membrane permeability [13]. It is even more challenging for developing peptide drugs for the use in the central nervous system (CNS), as the blood–brain barrier (BBB) is a huge obstacle for peptides to cross [14]. This can be seen clearly in the drug discovery of peptide-based opioid analgesics. Although many peptides have been identified as potent opioid receptor agonists in vitro, only a few can be used systemically to produce antinociception in vivo; the number of peptides that can produce CNS-mediated opioid analgesia is even less [7].

In our previous research, we identified CycloAnt, which is a cyclic peptide that exhibits mixed-functional MOR agonist/DOR antagonist and produces potent and efficacious antinociception in mice [15]. Intraperitoneal (i.p.) administration of CycloAnt produces a dose- and time-dependent antinociception with an ED_50_ of 0.7 mg/kg in the 55 °C warmwater tail-withdrawal assay (WWTW). Importantly, at high doses up to 15 times of the ED_50_ value (ED_50×15_), CycloAnt does not produce any respiratory depression, while chronic administration produces significantly reduced physical dependence, shown by naloxone-precipitated withdrawal testing [15]. In this study, we determine the binding affinity of CycloAnt at the three opioid receptors to verify its opioid receptor engagement. We carry out a computational study to predict the site of metabolism (SOM) as well as evaluate its stability in mouse serum and mouse liver microsomes. We also study the pharmacokinetics of CycloAnt and confirm its presence in the mouse brain. Investigation of its conformational dynamics using variant temperature ^1^H NMR experiment suggests that this beyond rule-of-5 (bRo5) [16,17] molecule behaves as a chameleonic peptide which can adjust its conformation in response to solvent change.

## 2. Results

### 2.1. Determination of Binding Affinity to Opioid Receptors

CycloAnt is a lariat peptide that acts in vivo as a bifunctional MOR agonist and DOR antagonist, which was confirmed in our previous study using opioid receptor individually knock-out (OP KO) mice [14]. In the current study, the binding affinities of CycloAnt to the three opioid receptors are determined using cell-based radioligand opioid receptor binding assays [18]. Using [^3^H]diprenorphine as the radioligand, we find that CycloAnt binds to the MOR and DOR with an affinity (*K*_i_) of 2.25 ± 0.44 and 7.85 ± 0.77 nM, respectively. At the KOR, CycloAnt at 1 μM inhibits 6% and at 10 μM inhibits 26% of [^3^H]diprenorphine binding. The in vitro binding affinity data indicate that CycloAnt selectively interacts with MOR and DOR, agreeing with its in vivo receptor engagement in OP KO mice determined in our previous study [15].

### 2.2. Evaluation of the Proteolytic Stability of CycloAnt

**Figure 1:**
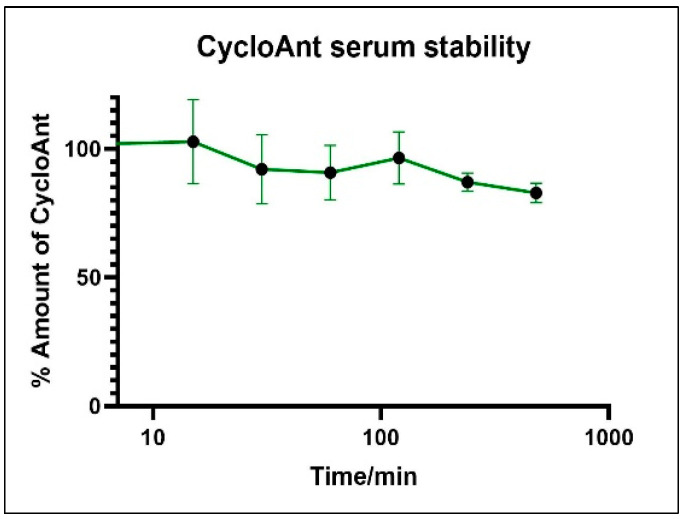
CycloAnt stability in mouse serum.

Proteolytic instability is a major hurdle limiting the development of peptide drug. As blood is a major source of proteases that degrade peptides, we evaluated the stability of CycloAnt in mouse serum. CycloAnt at a final concentration of 20 µM was incubated with mouse serum (SigmaAldrich) at 37 °C for 8 h. At 0, 15, 30, 60, 120, 240, and 480 min, the incubation was terminated by adding 2 volumes of ice-cold acetonitrile to precipitate the proteins in the sample. After centrifugation at 10,000 rpm, supernatant was collected. The processed sample was analyzed by liquid chromatography tandem mass spectrometry (LC-MS/MS). The percentage of CycloAnt remaining at each time point was calculated (Figure 1). During the 8 h frame, 82.3% of CycloAnt kept its initial form, indicating that CycloAnt is stable against enzymatic degradation in mouse serum.

### 2.3. Evaluation of the Metabolic Stability of CycloAnt

#### 2.3.1. Computational Prediction of P450 Site of Metabolism

**Figure 2:**
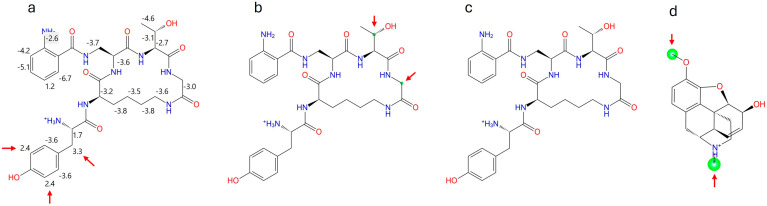
Maestro P450 Site of Metabolism (SOM) calculation of CycloAnt (**a**) intrinsic reactivity for 3A4; (**b**) overall SOM score for 2C9; (**c**) overall SOM score for 2D6. The SOMs observed are marked with a green circle. The size of the circle indicates the overall SOM score-the larger the circle, the better the score. (**d**) SOM calculation of codeine for 2D6 is utilized as a reference to indicate the size of green circle at the actual metabolic sites.

CycloAnt produces antinociception in mice with a short duration lasting 70 min, determined in our previous study [15]. As CycloAnt is stable against proteolytic degradation in serum, we speculated whether the short duration of action could be due to its quick metabolism by P450 enzymes. We first conducted a computational study to identify its potential sites of metabolism (SOMs) at 3A4, 2C9, and 2D6 using Maestro P450 Site of Metabolism (SOM). The intrinsic reactivity at 3A4 is calculated with a linear free energy approach based on the Hannett and Taft scheme [19]. Two atoms, C^β^ (3.3) and the C^3’^ (2.4) of the phenyl ring on the Tyr^1^ residue, were predicted to have weak intrinsic reactivity for 3A4 (Figure 2a). Induced-fit docking of CycloAnt to 2D6 combined with its intrinsic reactivity showed that 2 atoms, C^β^ of Thr^4^ and C^α^ of Gly^5^, have a low overall SOM score (small green circle in Figure 2b), indicating low reactivity at 2D6. Induced-fit docking of CycloAnt to 2C9 combined with its intrinsic reactivity did not identify any atom (no green circle in Figure 2c) that may be reactive at the heme site. The results of the computational prediction suggest that CycloAnt may not be a substrate of CYP3A4, 2C9, and 2D6.

#### 2.3.2. Evaluation of the Metabolic Stability of CycloAnt in Mouse Liver Microsomes

**Figure 3:**
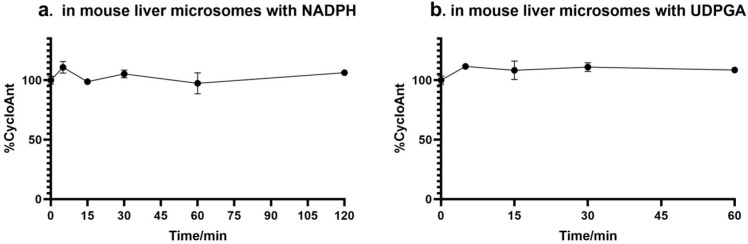
Stability of CycloAnt in mouse liver microsomes. The percentage of CycloAnt remaining after incubation with liver microsomes at 37 °C (**a**) in the presence of NADPH; (**b**) in the presence of UDPGA.

The metabolic stability of CycloAnt was then evaluated in mouse liver microsomes. CycloAnt at a final concentration of 3.07 μM was mixed with 0.5 mg/mL liver microsomes in the presence of 1 mM of NADPH and incubated at 37 °C for 2 h [20]. At 0, 5, 15, 30, 60, and 120 min, incubation mixtures were withdrawn and processed. The supernatant was analyzed by LC-MS/MS. The percentage of CycloAnt remaining at each time point was calculated (Figure 3a). During the 120 min incubation window, CycloAnt was observed to have no significant concentration change, indicating that CycloAnt was stable against CYP450 metabolism in mouse liver microsomes in vitro. This result also validates the prediction of the computational SOM simulation.

Next, we examined the level of glucuronidation of CycloAnt in mouse liver microsomes using a method described elsewhere [21]. CycloAnt at a final concentration of 3.07 μM was premixed with 0.5 mg/mL liver microsomes and a channel-forming peptide antibiotic alamethicin at 0 °C. After 15 min, the glucuronidation was initiated by adding the UDP-glucuronosyl transferases (UGTs) substrate uridine-5’-diphospho-α-D-glucuronic acid (UDPGA) to a final concentration of 5 mM and the mixture was incubated at 37 °C [19]. At 0, 5, 15, 30, and 60 min, the incubation mixture was withdrawn and processed. The supernatant was collected and analyzed by LC-MS/MS. The percentage of the remaining CycloAnt was calculated, and the results show that CycloAnt was stable against glucuronidation over the 60 min incubation period, indicating that CycloAnt was not metabolized by UGTs as well (Figure 3b).

#### 2.3.3. Examination of the Possible Metabolites Using Mass Spectrometry (MS)

To verify if CycloAnt could be metabolized in vivo, we looked for the possible metabolites (Scheme S1) in a blood sample collected from mice 15 min after i.p. administration of CycloAnt at a dose of 10 mg/kg. The proteins in the mouse plasma were precipitated by ice-cold acetonitrile containing internal standard. Following centrifugation, the supernatant was collected, and analyzed by LC-MS/MS. A total ion current (TIC) scan was conducted for the mass-to-charge ratio (*m*/*z*) in the range of 300–1500 for all ions, followed by the targeted narrow range scans. The scans in the range of 650–690 and 325–346 are aimed at detecting the potential [M + 1]^+^ and [M + 2]^2+^ ions from the Phase I metabolites, the enzymatically cleaved peptides, and intact CycloAnt, respectively (Figure S1 and Figure 2); and the scans in the range of 843–852 and 422–428 are for detecting the potential [M + 1]^+^ and [M + 2]^2+^ ions from the Phase II glucuronidation, respectively (Figure S3). The [M + 1]^+^ and [M + 2]^2+^ ions were then extracted from the narrow range scans for the potential enzymatically cleaved products (Figure S1), the Phase I metabolites (Figure S2), and the Phase II UGT metabolites (Figure S3). The relative abundance of the tallest peak was assigned as 100. The data show that the m/z intensity of any possible metabolites is within the range of 1E3 to 1E4 (Figures S1–S3, top and middle panels); however, the *m*/*z* intensity of CycloAnt in the plasma sample reaches 1E7 (Figure S1–S3, bottom panel). The MS data suggest that any metabolites generated from the potential metabolic reactions should have concentrations 10^3^- to-10^4^-fold lower than CycloAnt. In combination with the results from the in vitro stability study, we are confident that CycloAnt is stable in plasma and resistant to liver metabolism in mice.

### 2.4. Evaluation of Pharmacokinetics of CycloAnt in Mice

CycloAnt is a cyclic peptide that exhibits mixed-functional MOR agonist/DOR antagonist and produces potent and efficacious antinociceptive effects in mice. We have reported that i.p. preadministration of CycloAnt to MOR-KO mice dose-dependently blocked the antinociceptive effect produced by a DOR agonist, SNC-80, administrated intracerebroventricularly (i.c.v.) [15]. This effect indicates that CycloAnt enters into the brain to antagonize the antinociceptive effect of SNC-80. As CycloAnt is a potent MOR agonist in vitro and is stable in mouse serum and liver microsomes, we believe that the pharmacological effects produced by i.p. administrated CycloAnt are not caused by its hydrolyzed fragments or metabolites, but the intact CycloAnt molecule.

To evaluate the amount of CycloAnt in mouse brain, we performed a pharmacokinetic experiment in mice [22]. Mice were given CycloAnt at 3 mg/kg i.p.; blood was collected at 0, 15, 30, and 60 min post-injection. Immediately after the blood was collected, the mouse was intracardially perfused through the left ventricle with 20 mL PBS. The whole brain was isolated and homogenized. The blood and brain samples were processed by protein precipitation and centrifugation. The supernatants of the blood and brain samples were collected and analyzed by LC-MS/MS.

**Figure 4:**
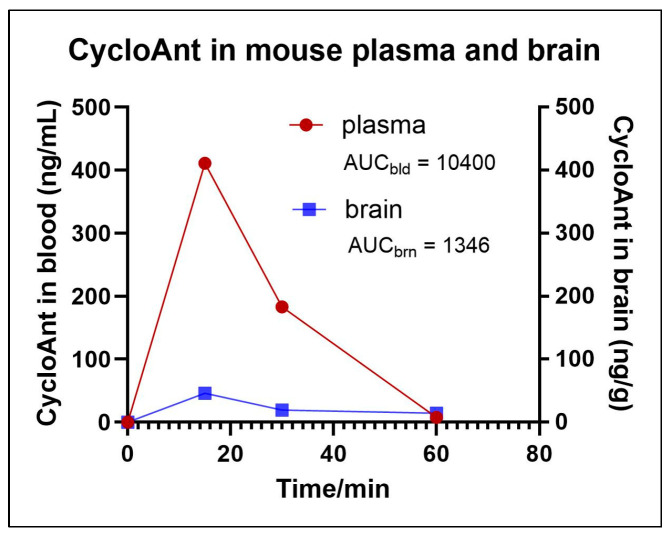
The amount of CycloAnt detected in mouse plasma and brain.

The LC-MS/MS detection shows that CycloAnt is present in blood and brain; at 15 min post-injection it reaches the maximum concentrations in blood as well as in the brain. The maximum concentration of CycloAnt in mouse plasma (C_max,pl_) was calculated to be 411 ng/mL and the maximum concentration of CycloAnt in brain (C_max,br_) was 46 ng/g, or 70 nM, assuming that the density of brain tissue is 1 g/mL (Figure 4). This study confirmed that CycloAnt penetrated the BBB after systemic administration. The brain-to-plasma ratio of CycloAnt is 11.5%, determined by the ratio of the areas under the curves (AUC), which is a high extent of BBB transport in terms of a peptide [23].

### 2.5. NMR Characterization of CycloAnt for Its Conformational Plasticity with Environmental Change

**Figure 5:**
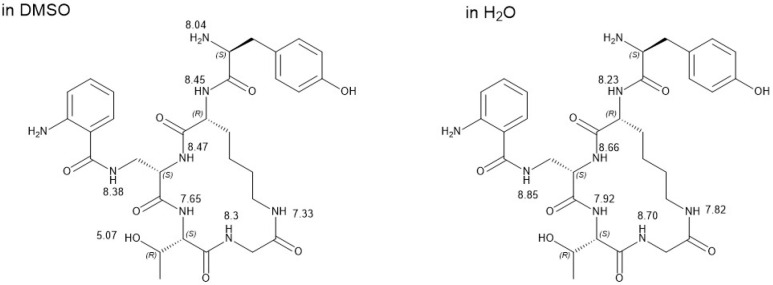
^1^H NMR peak assignment of CycloAnt recorded in DMSO and H_2_O at 298 K.

CycloAnt has 10 HB donors, 27 rotating bonds, a molecular weight of 654.7, and a cLogP value of −0.7323. It is a bRo5 molecule that classically is considered membrane impermeable. Even though its backbone is constrained, limiting the possible rotations around the peptide bonds, it still adopts multiple conformations. To understand how CycloAnt penetrates the BBB, we conducted variant-temperature NMR experiments in solvents of varying polarity, i.e., water (dielectric constant, ε ≈ 80) and less polar solvent DMSO (ε = 47) (Figures S4 and S5), to assess its conformation in the two solvents. The dielectric constant of DMSO is between water (ε ≈ 80) and nonpolar interior of membrane (ε ≈ 2–3); therefore, it is suggested to mimic the interface between water and membrane, or where peptide initially interacts with membrane [24]. The ^1^H NMR peaks recorded in the two solvents at 298 K were assigned to each amide proton (H^N^) on CycloAnt (Figure 5). The temperature shift coefficient (Δδ/ΔT) of each H^N^ was then calculated (Table 1) to evaluate their involvement in forming an intramolecular hydrogen bond (HB) in the two solvents (Figures S6 and S7).

The temperature shift coefficient of each amide H^N^ measured in water differed significantly from that in DMSO. When CycloAnt is in DMSO, all H^N^s have a higher temperature shift coefficient (less negative) value than in water. Specifically, in DMSO, all amide H^N^s have a Δδ/ΔT value equal to or larger than −4.0 ppb/K, with the H^N^ of Thr^4^ and the H^αN^ of Dap^3^ being −1.3 and −3.1 ppb/K. In water, the Δδ/ΔT values of the H^N^s of Thr^4^ and the H^αN^ of Dap^3^ are −3.9 ppb/K, but the rest of the H^N^s all have a much lower Δδ/ΔT value in the range of −6.3 to −8.0 ppb/K.

## 3. Discussion

Peptides have a large polar surface area with a high number of free rotatable bonds; they are classic bRo5 molecules unfavored for membrane permeation. However, peptides may utilize various mechanisms to penetrate the membrane, such as endocytosis and direct membrane translocation, or active transport [23,25,26,27,28]. Some natural cyclic peptides, e.g., cyclosporin A, have high membrane permeability. Cyclosporin A has multiple N-methylated amide bonds; N-methylation improves its hydrophobic and passive membrane permeability [25]. Cyclosporin A can also adjust its conformation in response to environmental changes. This chameleonic property facilitates the membrane translocation of cyclosporin A [27]. Cyclic pentapeptide cilengitide (cyclo[RGDfNMeV]) and its amide-to-ester substituted analogs, which are similar in size to CycloAnt, have also been reported to display conformational plasticity [29].

Conformationally plasticity is a key determinant of membrane permeability of cyclic peptides [30]. Our variant temperature NMR experiments confirmed that the temperature shift coefficient of each amide H^N^ of CycloAnt changed with varying solvent polarity/environment. Temperature shift coefficient has been used to distinguish the hydrogen-bonded or nonhydrogen-bonded protons in protein and peptide. Using a cutoff value of −4.5 ppb/K for hydrogen-bonded amide [31], in DMSO, all the amide H^N^s of CycloAnt are intramolecularly hydrogen bonded and the H^N^ of Thr and the H^αN^ of Dap are locked in strong intramolecular HBs, while in water, only H^N^ of Thr and the H^αN^ of Dap are intramolecularly hydrogen bonded. The other 4 amide H^N^s are exposed to the solvent without forming intramolecular HBs. The difference of the amide involvement in the intramolecular hydrogen bonding clearly indicates that CycloAnt adopts different conformations in DMSO and water, showing the chameleonic behavior. In water, these amide H^N^s are void of intramolecular HBs, suggesting that CycloAnt has an extended conformation with most amide H^N^s being exposed to water, making it water soluble. In a non-/less polar environment, all amide H^N^s of CycloAnt are shielded by involving intramolecular HBs, thus driving CycloAnt into a compact conformation [32]. The chameleonic property explains how CycloAnt, a lariat opioid peptide, can adjust its conformation in response to the solvent/environmental change, which helps it permeate the BBB to produce CNS-mediated antinociception.

Opioid peptides have been accepted as promising candidates for the development of safer and more efficacious analgesics owing to their unique advantages over small molecules in the context of safer opioid analgesics, such as simultaneously engaging the receptors at the orthosteric binding pocket and the extracellular loops, activating the receptors at both plasma membrane and endosomes, etc. [7]. However, developing opioid peptides for CNS application is a significant challenge since peptides are generally subjected to enzymatic degradation and impermeable to BBB. CycloAnt, a lead cyclic peptide synthesized and identified in our drug discovery research, can be administrated systemically to produce potent and efficacious CNS-mediated analgesia without significant opioid receptor-mediated side effects. Our studies on proteolytic stability, metabolic stability, and pharmacokinetics confirm that CycloAnt is not only stable in mouse serum and liver microsomes, but also crosses the BBB in mice. Our conformational complexity study indicates that CycloAnt adjusts its conformation in response to the environmental/solvent change. Though this study did not eliminate the possibility that CycloAnt may also employ the active transport mechanism to enter the brain, its chameleonic property can certainly benefit its BBB permeation. Further modification of this proteolytically and metabolically stable, brain-permeable cyclic peptide may grant opportunity to develop cyclic peptides with higher stability and BBB permeability for the development of safer opioid analgesics.

## 4. Materials and Methods

**Materials**. CycloAnt was synthesized in house by the method described in [15]. Mouse serum, NADPH, and uridine 5’-diphosphoglucuronic acid trisodium salt were purchased from SigmaAldrich (St. Louis, MO, USA). Mouse liver microsomes were purchased from Gibco and alamethicin was purchased from Fisher Scientific (Waltham, MA, USA). [^3^H]diprenorphine was purchased from Revvity (Waltham, MA, USA).

LC-MS/MS was recorded on Waters Quattro Premier XE triple quadrupole MS system with a Waters Acquity UPLC. NMR spectra were recorded on a Bruker Avance III-HD 400 instrument (Germany) at 400 MHz for ^1^H NMR. NMR chemical shifts are expressed in ppm relative to internal solvent peak, and coupling constants were calculated in hertz.

**Opioid receptor radioligand binding assay**. Membranes of CHO cells stably expressing the rat MOR, mouse DOR, or human KOR were used in the experiments. [^3^H]diprenorphine (PerkinElmer, Waltham, MA, at the time of experiment calculated to have specific activity 35 Ci/mmol) was used as the radioligand for the MOR, DOR, and KOR. Competition inhibition of [^3^H]diprenorphine (0.3–0.4 nM) binding to the MOR or DOR was conducted in 50 mM Tris-HCl buffer (pH 7.4) with an adequate number of cell membranes for each receptor and 10^−1^ to 10^−5^ M of CycloAnt. The amounts of cell membranes used were 10–30 μg membrane protein/mL, which were sufficient to give 2000–2500 dpm specific binding. Nonspecific binding, defined as the binding in the presence of 10 μM naloxone, was <300 dpm. The numbers of the receptors were 20–30 fmole receptor/mL in the assay. The mixture was incubated at room temperature for one hour. Binding reactions were terminated by rapid filtration under vacuum over GF/B filters presoaked in 0.1% polyethyleneimine, and the bound radioactivity was counted by liquid scintillation counting. CycloAnt was examined for its competitive inhibition of [^3^H]diprenorphine (0.3–0.4 nM) to the KOR at two concentrations of 1 and 10 µM. Binding data were analyzed using GraphPad Prism software (version 10.2.3) and IC_50_ values (the concentration necessary to inhibit 50 percent of radioligand binding) and *K*_i_ values were calculated. Data were obtained from 3 independent experiments, each in duplicate.

**Stability in mouse serum**. CycloAnt was dissolved in pH 7.2 phosphate buffer saline (PBS). A total of 175 µL of CycloAnt at a final concentration of 20 µM was mixed with 2.625 mL of 100% mouse serum (SigmaAldrich) and incubated at 37 °C. The mixture was analyzed over a time course of 8 h. At the time points of 0, 15, 30, 60, 120, 240, and 480 min, 150 µL of sample were withdrawal and the reaction was terminated with 300 μL of ice-cold acetonitrile containing internal standard (IS, Tyr-[D-Lys-Phe-Tyr-Gly] at 200 ng/mL). The mixture was centrifuged at 10,000 rpm at 4 °C for 10 min. An aliquot of 300 μL supernatant was collected and dried under vacuum, followed by reconstitution of the sample in 20% acetonitrile. The sample was then centrifuged at 13,000 rpm at 4 °C for 5 min to remove the lipid content. The supernatant was collected for LC-MS/MS analysis. The percentage of peptide remaining at each time point was calculated relative to 0 min. The experiment was performed in duplicate.

**Metabolism by CYP450 in liver microsomes**. CycloAnt dissolved in pH 7.2 PBS was diluted to 8 μM. Six hundred microliters of CycloAnt were incubated with 800 μL of 0.975 mg/mL mouse liver microsomes and 160 μL of 10 mM NADPH at 37 °C for 0, 5, 15, 30, 60, and 120 min. The final concentrations of CycloAnt, mouse liver microsomes, and NADPH in the incubation mixture were 3.07 μM, 0.5 mg/mL, and 1 mM, respectively. At the designated time points, 100 μL of the incubation mixture were withdrawn and mixed with 200 μL ice-cold acetonitrile containing internal standard (IS, Tyr-[D-Lys-Phe-Tyr-Gly]), followed by centrifugation at 10,000× *g* at 4 °C for 10 min. An aliquot of 250 μL of supernatant was collected and dried in vacuum. The dried sample was reconstituted in 100 μL of 20% acetonitrile and stored at −20 °C before analyzed by LC-MS/MS on the next day. The experiment was performed in duplicate.

**Metabolism by UDP-glucuronosyl transferases (UGTs) in liver microsomes**. CycloAnt dissolved in pH 7.2 PBS was diluted to 8 μM. Six hundred microliters of diluted CycloAnt, 800 μL of 0.975 mg/mL mouse liver microsomes, and 14 μL of 0.5 mM alamethicin containing 10 mM MgCl_2_, were mixed and preincubated on ice for 15 min. The reaction was then initiated by adding 74 μL of 100 mM uridine-5’-diphospho-α-D-glucuronic acid (UDPGA) and incubated at 37 °C for 0, 5, 15, 30, and 60 min. At the designated time points, 100 μL of the incubation mixture were withdrawn, followed by adding 200 μL ice-cold acetonitrile containing 200 ng/mL of an internal standard (IS, Tyr-[D-Lys-Phe-Tyr-Gly]). The sample was then centrifuged at 10,000× *g*, 4 °C for 10 min. An aliquot of 250 μL of supernatant was withdrawn and dried in vacuum. The dried sample was reconstituted in 100 μL of 20% acetonitrile and stored at −20 °C before analyzed by LC-MS/MS on the next day. The experiment was performed in duplicate.

**Pharmacokinetic study. Standard curve.** Following euthanasia, mouse blood was collected in a heparin anticoagulation tube and the whole brain was isolated. For determination of the standard curve in mouse blood, 300 μL of blood was spiked with 0, 10, 100, and 1000 ng of CycloAnt, respectively, followed by adding 600 μL of ice-cold acetonitrile containing the internal standard (IS, Tyr-[D-Lys-Phe-Tyr-Gly]) at 200 ng/mL. The blood samples were thoroughly mixed and centrifuged at 10,000 rpm at 4 °C for 10 min. An aliquot of 600 μL of supernatant from each sample was collected and dried under vacuum, followed by reconstitution of the dried blood sample in 100 μL 20% acetonitrile. The reconstituted sample was centrifuged at 13,000 rpm, 4 °C for 5 min to remove the lipid content. The supernatant was collected for LC-MS/MS analysis. For mouse brain, the brain was homogenized with 250 μL of PBS at 0 °C, followed by adding 1 mL of ice-cold acetonitrile containing the IS. The brain sample was then spiked with 0, 10, 100, and 1000 ng of CycloAnt, respectively, and thoroughly mixed and centrifuged at 10,000 rpm, 4 °C for 10 min. An aliquot of 1000 μL of supernatant was collected and dried under vacuum, followed by adding 100 μL 20% acetonitrile to reconstitute the dried brain sample. The reconstituted sample was mixed and centrifugating at 13,000 rpm, 4 °C for 5 min to remove the lipid content. The supernatant was collected and analyzed by LC-MS/MS.

The peak areas of CycloAnt and IS were recorded; the relative measured concentration of CycloAnt was calculated using the ratio of CycloAnt peak area vs. IS peak area. A standard concentration curve (Figure S8) was then generated for CycloAnt in the matrix of blood and brain, respectively.

**Pharmacokinetics.** Eight-week-old male C57BL/6 mice (Jackson Laboratories, Bar Harbor, ME, USA) were used for pharmacokinetic studies. CycloAnt was given to mice (*n* = 2) *i.p*. at a dose of 3 mg/kg. At 15, 30, and 60 min post-administration, mouse blood was collected in a heparin anticoagulation tube. After blood collection, the mouse was intracardially perfused through the left ventricle with 20 mL PBS, followed by isolation of the whole brain. To process the blood sample, 300 μL blood was mixed with 600 μL of ice-cold acetonitrile containing the IS at 200 ng/mL, followed by centrifugation at 10,000 rpm, 4 °C for 10 min. To process the brain sample, 250 μL of ice-cold PBS was added to the isolated brain and homogenized at 0 °C, followed by mixing with 1 mL of ice-cold acetonitrile containing the IS. The brain sample was then centrifuged at 10,000 rpm, 4 °C for 10 min. The supernatants were collected from the blood sample (600 μL) and the brain sample (1 mL), respectively, and dried under vacuum. The blood and brain samples were reconstituted in 100 μL of 20% acetonitrile. The samples were then centrifuged at 13,000 rpm, 4 °C for 5 min to remove the lipid content. The supernatants were collected from the blood and brain samples and analyzed by LC-MS/MS.

**Computational prediction of P450 Site of Metabolism**. The P450 Site of Metabolism prediction was performed using Maestro (Schrödinger). CycloAnt was prepared in LigPrep using OPLS4 force field. Induced fit docking plus intrinsic reactivity were performed at 2C9 and at 2D6. Intrinsic reactivity calculation was performed at 3A4.

**Variant Temperature NMR study**. CycloAnt was dissolved in DMSO-*d*6 at a concentration of 10 mg/mL (15.3 mM) and the ^1^H NMR experiments were conducted at 7 temperature points at 298, 305, 310, 315, 320, 325, and 330 K. The chemical shift of DMSO (2.50 ppm) was used as a standard for calibration. For the ^1^H NMR experiment in water, CycloAnt was dissolved in a mixture of 85%H_2_O:10%D_2_O:5%DMSO-*d*6 at a concentration of 10 mg/mL (15.3 mM). The experiments were carried out at 283 K with an elevation of 5 K at each point to the highest temperature of 303 K. The chemical shift was calibrated using DMSO as a standard, which has a chemical shift of 2.71 ppm in water [33].

## Figures and Tables

**Table 1:** Temperature coefficient (Δδ/ΔT) of amide proton H^N^ of CycloAnt in DMSO and H_2_O.

Δδ/ΔT (ppb/K)	H^N^ Gly^5^	H^N^ Thr^4^	H^αN^ Dap^3^	H^γN^ Dap^3^	H^αN^ D-Lys^2^	H^εN^ D-Lys^2^
In DMSO	−3.8	−1.3	−3.1	−3.7	−4.0	−3.9
In H_2_O	−6.8	−3.9	−3.9	−8.0	−6.3	−6.7

## Data Availability

Data are contained within the article and the Supplementary Materials.

## Supplementary Materials

The following supporting information can be downloaded at: https://www.mdpi.com/article/10.3390/ijms252111389/s1.
