# Supplementary material for: Conformational Plasticity Enhances the Brain Penetration of a Metabolically Stable, Dual-Functional Opioid-Peptide CycloAnt
Source: Int J Mol Sci. 2024 Oct 23;25(21):11389. doi: 10.3390/ijms252111389 (PMC11546339; doi:10.3390/ijms252111389)
Supplement: Supplementary file 1 [file ijms-25-11389-s001.zip › ijms-3224100-supplementary.pdf]

Supplementary Materials

Conformational Plasticity Enhances the Brain Penetration of a Metabolically Stable, Dual-Functional Opioid-Peptide CycloAnt

Table of Contents

1. Potential metabolites of CycloAnt and the extracted ions out of the total ion current (TIC) of plasma collected from mouse administrated CycloAnt
2. TOCSY spectra of CycloAnt in DMSO-d6 and H_2O
3. ^1H NMR spectra at varying temperatures in DMSO-d6 and H_2O
4. ^1H NMR Chemical shift of amide proton at varying temperature and temperature-dependent chemical shift plot in DMSO and H_2O
5. CycloAnt standard detection curves in blood and brain

1. Potential metabolites of CycloAnt and the extracted ions out of the TIC of plasma collected from mouse administrated CycloAnt

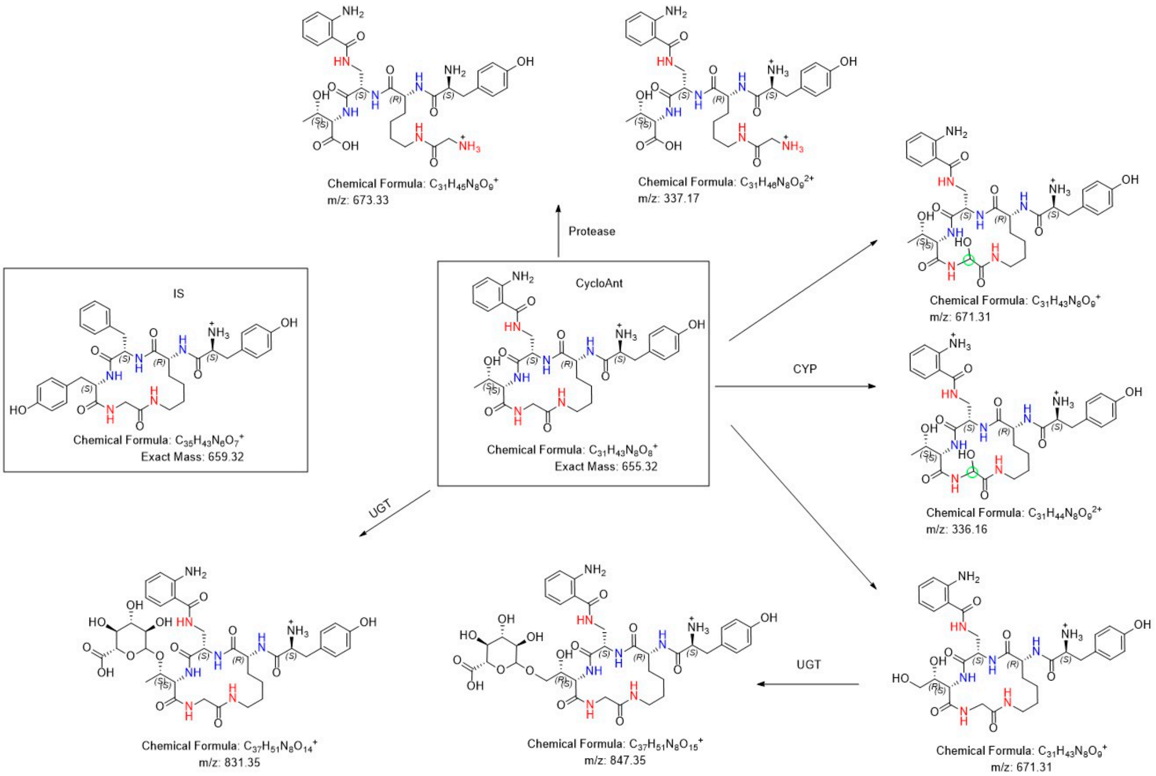

Scheme S1. Potential metabolites of CycloAnt by enzymatic cleavage, CYPs, and UGT transformation.

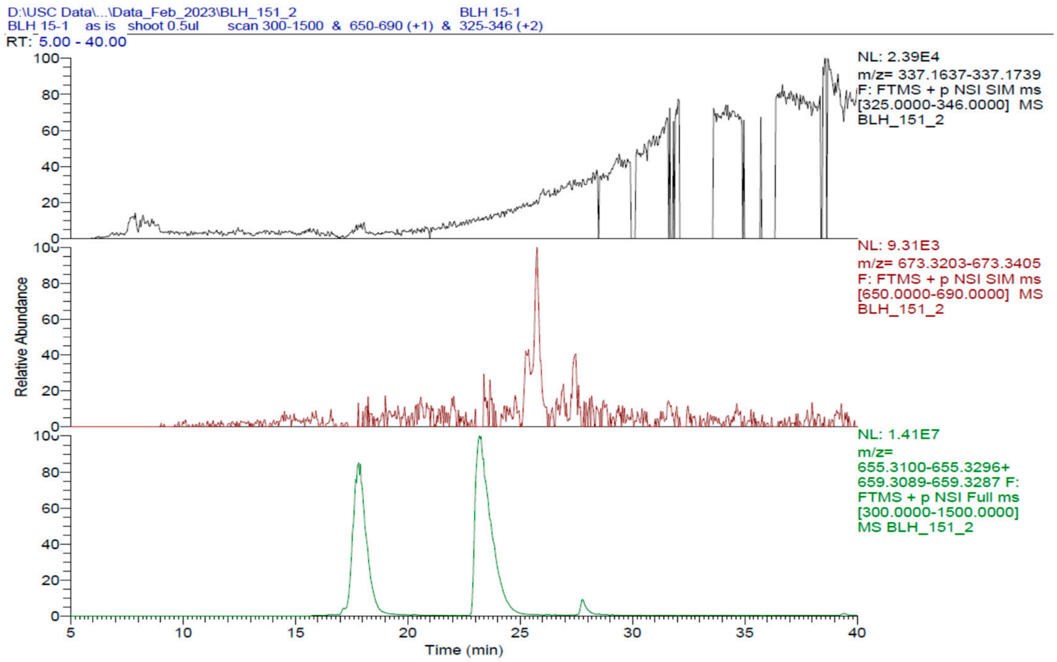

Figure S1. Extracted ions for enzymatic cleavage products and CycloAnt in the plasma collected from mice administrated CycloAnt. Top: [M+2] ion of cleaved product; middle: [M+1] ion of cleaved product; bottom: CycloAnt [M+1] ion at 17.87 min and the internal standard (IS) [M+1] ion at 23.20 min.

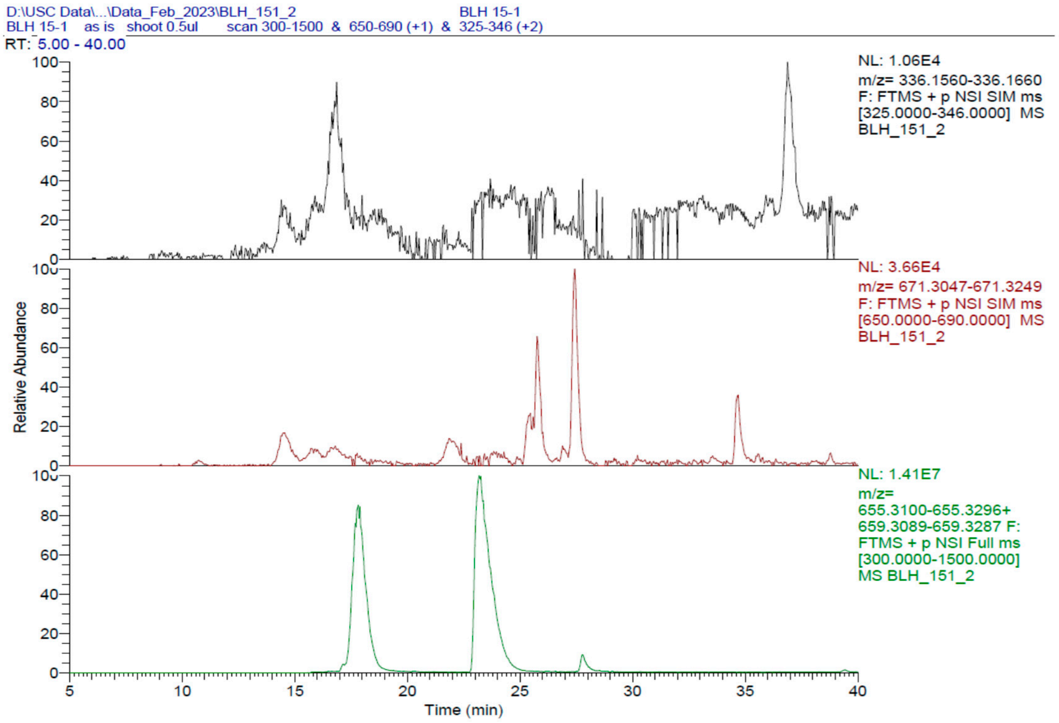

Figure S2. Extracted ions for Phase I metabolites and CycloAnt in the plasma collected from mice administrated CycloAnt. Top: [M+2] ion of metabolites; middle: [M+1] ion of metabolites; bottom: CycloAnt [M+1] ion at 17.87 min and the IS [M+1] ion at 23.20 min.

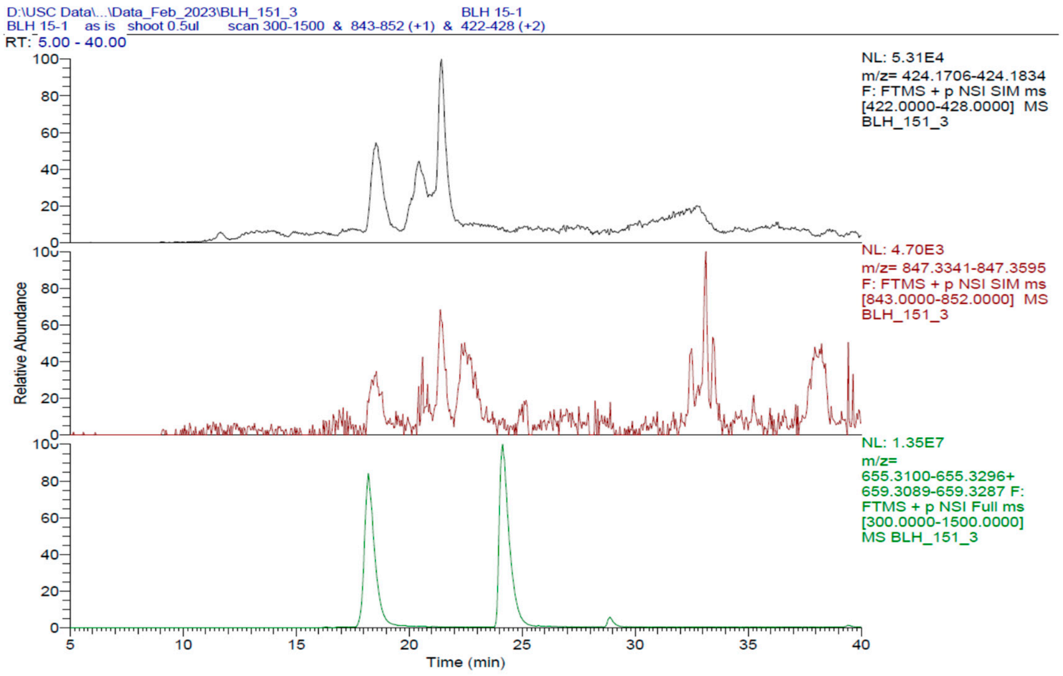

Figure S3. Extracted ions for Phase II metabolites and CycloAnt in the plasma collected from mice administrated CycloAnt. Top: [M+2] ion of metabolites; middle: [M+1] ion of metabolites; bottom: CycloAnt [M+1] ion at 17.87 min and IS [M+1] ion at 23.20 min.

2. TOCSY Spectra of CycloAnt in DMSO-d6 and H_2O
   In DMSO

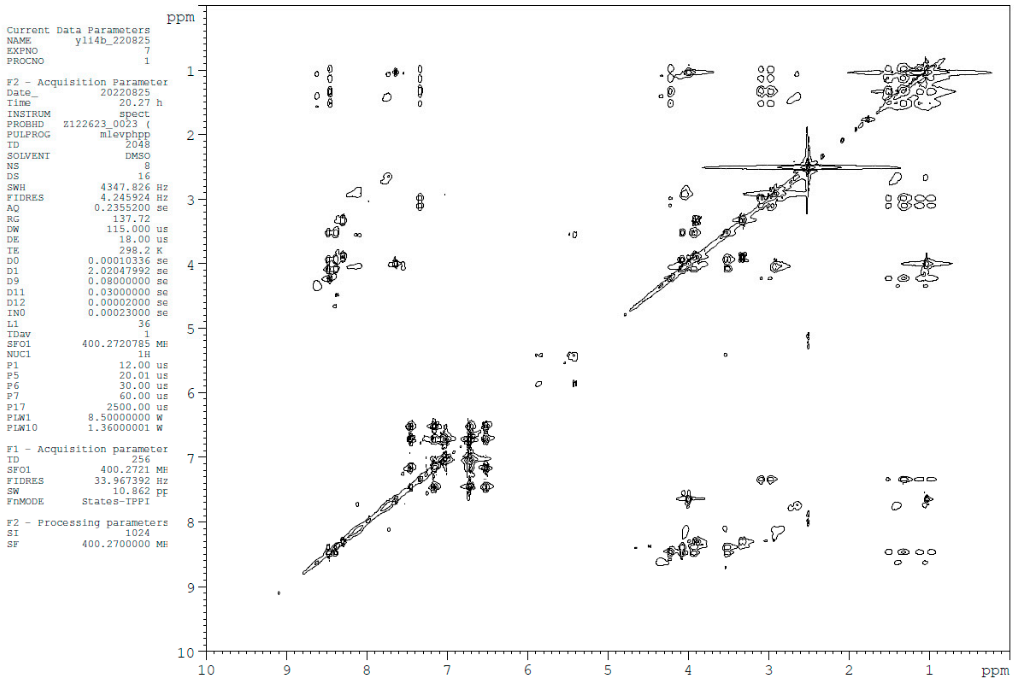

In H_2O

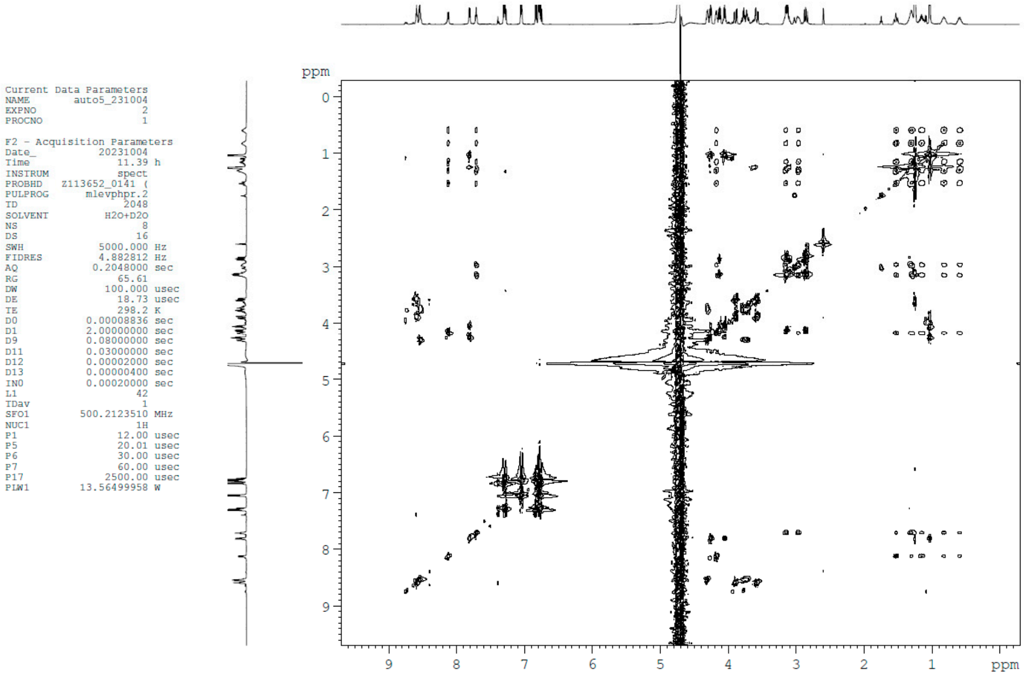

Figure S4. TOCSY Spectra of CycloAnt in DMSO-d6 (top) and H_2O (bottom)

3. ^{1}H NMR spectra at different temperatures

In DMSO

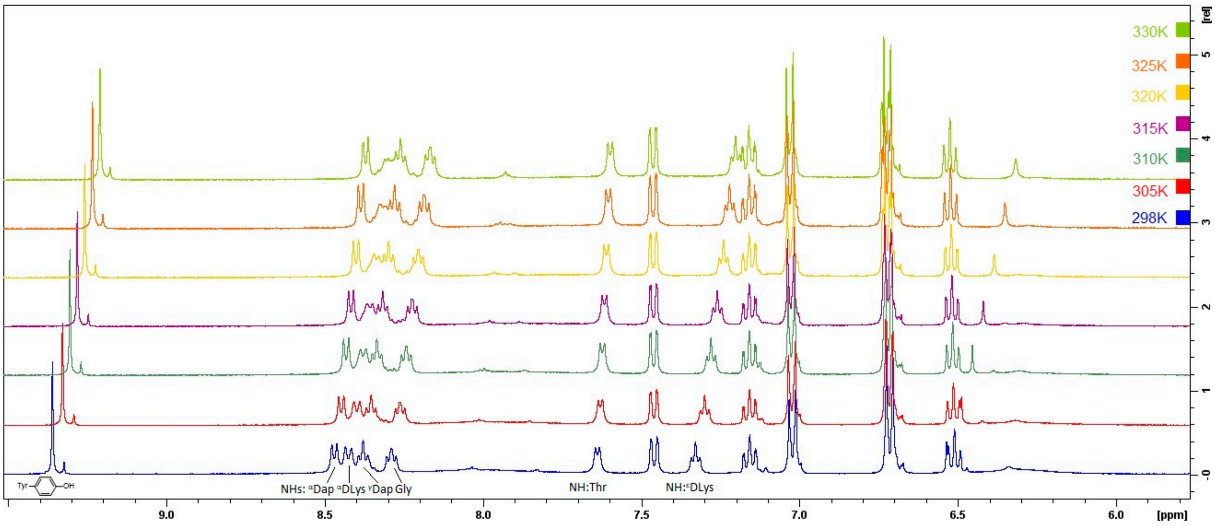

In H_2O

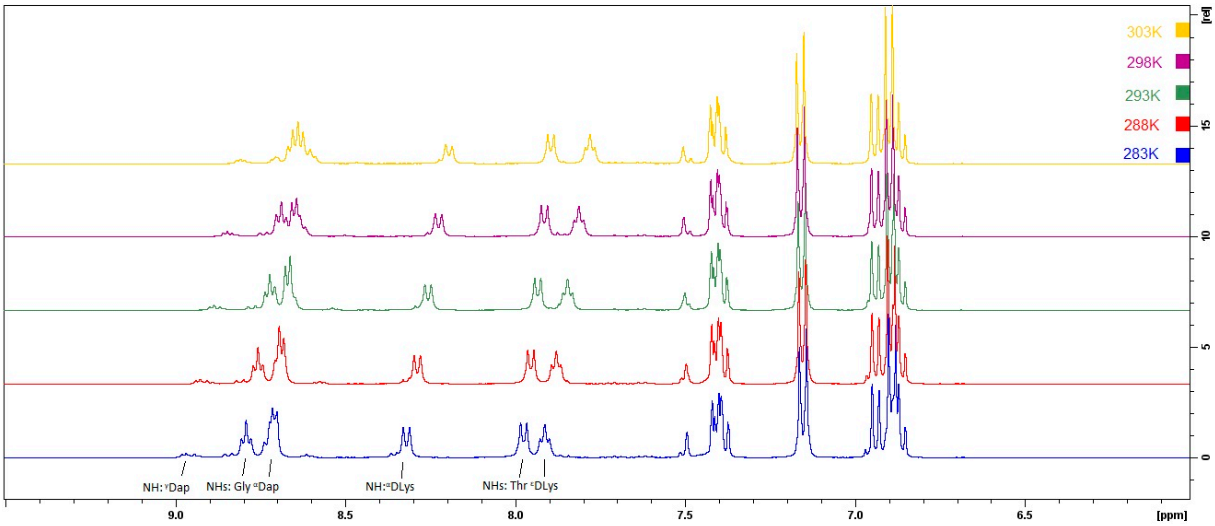

Figure S5. ^{1}H NMR spectra at different temperatures in DMSO (top) and in H_2O (bottom)

4. ^{1}H NMR Chemical shift of amide proton in DMSO and H_2O at varying temperature and plot of temperature-dependent chemical shift

In DMSO-d6

Temperature (K)	Gly NH	Thr NH	Dap NH(α)	Dap-NH(γ)	D-Lys NH(α)	D-Lys NH(ε)
298	8.2898	7.6391	8.4708	8.3797	8.4263	7.3292
305	8.2624	7.6302	8.4488	8.3542	8.399	7.3005
310	8.2433	7.6239	8.4332	8.3316	8.3802	7.2803
315	8.2242	7.6177	8.4177	8.3175	8.3573	7.2605
320	8.2052	7.6112	8.4022	8.2989	8.3378	7.2408
325	8.1862	7.6051	8.3868	8.2799		7.2217
330	8.168	7.5986	8.3716	8.2613	8.3057	7.2029
Δδ/ΔT(ppb/K)	-3.8	-1.3	-3.1	-3.7	-4	-3.9

Temperature-dependent chemical shift plot

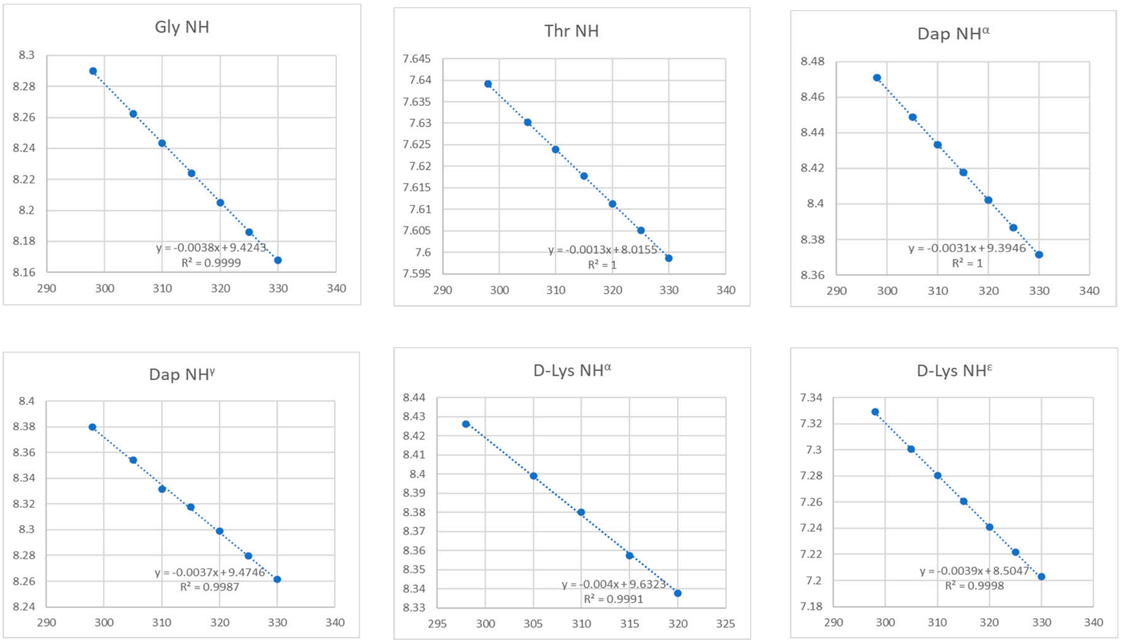

Figure S6. Plots of the temperature-dependent chemical shift in DMSO

In H_2O

Temperature (K)	Gly NH	Thr NH	Dap NH(α)	Dap-NH(γ)	D-Lys NH(α)	D-Lys NH(ε)
283	8.794	7.976	8.711	8.975	8.321	7.914
288	8.758	7.956	8.688	8.935	8.29	7.881
293	8.725	7.936	8.671	8.895	8.258	7.848
298	8.69	7.917	8.652	8.855	8.227	7.814
303	8.657	7.897	8.632	8.816	8.196	7.781
Δδ/ΔT(ppb/K)	-6.8	-3.9	-3.9	-8	-6.3	-6.7

Temperature-dependent chemical shift plot

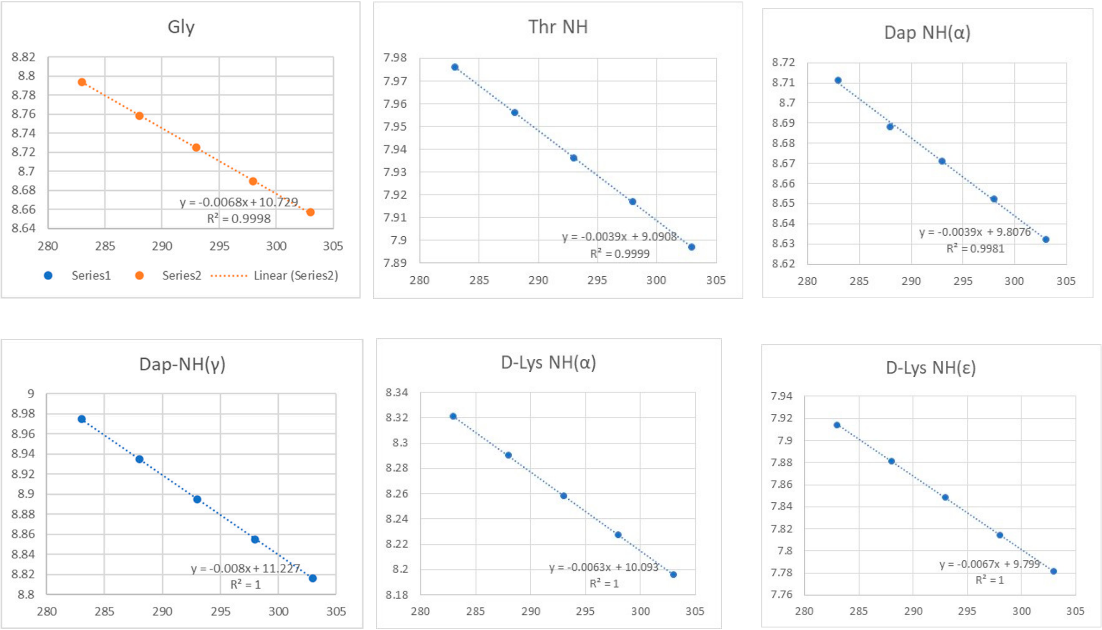

Figure S7. Plots of the temperature-dependent chemical shift in H_2O

5. CycloAnt standard detection curve
CycloAnt in blood

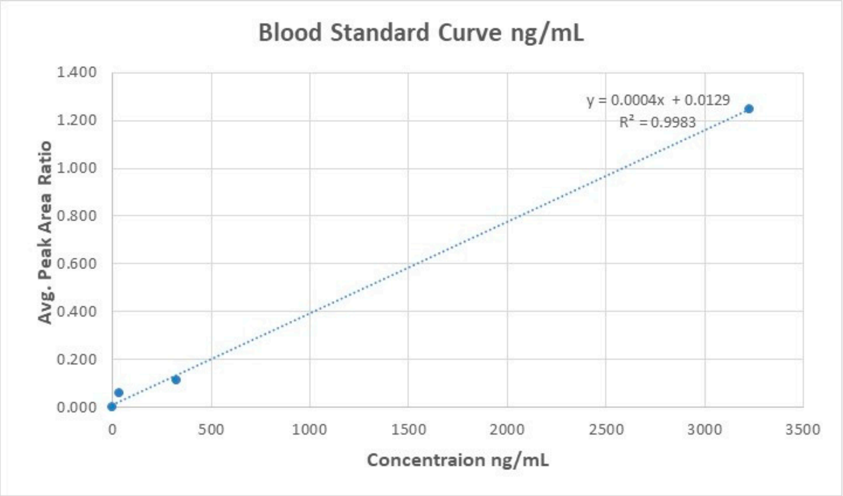

CycloAnt in brain

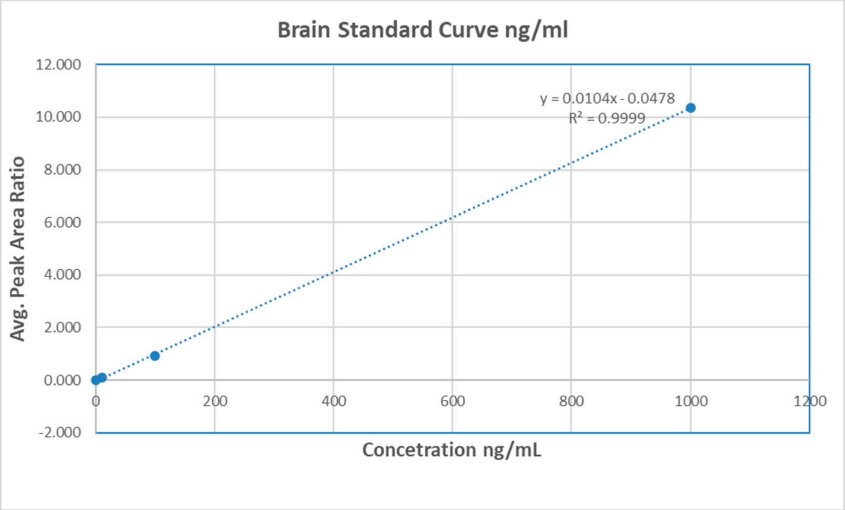

Figure S8. Plots of the standard detection curves of CycloAnt in mouse plasma (top) and brain (bottom)
